# The association between hypoglycemia and hospital use, food insufficiency, and unstable housing conditions: a cross-sectional study among patients with type 2 diabetes in Sudan

**DOI:** 10.1186/s13104-019-4145-z

**Published:** 2019-02-28

**Authors:** Hyder Osman Mirghani

**Affiliations:** 1 Department of Internal Medicine, Faculty of Medicine, University of Tabuk, Tabuk, Saudi Arabia; 2 Faculty of Medicine, University of Tabuk, PO Box 3378, Tabuk, 51941 Saudi Arabia

**Keywords:** Hypoglycemia risk, Food insufficiency, Type 2 diabetes, Sudan

## Abstract

**Objectives:**

Hypoglycemia is associated with mortality and healthcare utilization. We aimed to assess hypoglycemia risk and Hospital use among Sudanese patients with type 2 diabetes.

**Results:**

One hundred and fifty-nine patients with type 2 diabetes attending a diabetes center in Omdurman, Sudan during the period from June to September 2018were approached. A structured questionnaire based on hypoglycemia risk and Hospital use, Fasting plasma glucose (FPG) and the glycated hemoglobin (HbA_1c_) was used to interview the patients. Participants (age 58.13 ± 9.96 years), 4.4%, 14.5%, and 81.1% were at high, moderate, and low hypoglycemia respectively, 66% reported food insufficiency, while 15.1% had unstable housing conditions. No relationship was evident between the hypoglycemia risk, gender, unstable housing conditions, food insufficiency, fasting plasma glucose,HbA_1c_, and the duration since the diagnosis of diabetes. A considerable number (18.9%) of Sudanese patients with diabetes were at moderate/high risk of hypoglycemia and Hospital use, including hypoglycemia risk and hospital use assessment in the holistic care of diabetes are recommended.

## Introduction

Diabetes mellitus is a global health burden causing a lot of mortality and morbidity; the disease has attained an epidemic proportion. Currently, 285 million are affected, and this number is projected to reach 438 million by the year 2030, in Sudan 10.9% are affected by the disease [1].

Hypoglycemia is the most common serious complication of diabetes medications and may be a treatment-limiting factor. Hypoglycemia although preventable could incur a substantial burden on the patients, healthcare systems, and the community as a whole [2].

The American Diabetes Association recommended glycated hemoglobin of < 7 to reduce microvascular complications [3]. A J-shaped relationship was found between the glycated hemoglobin and all-cause mortality with an increasing death at both high and low HbA1c [4], a recent study showed an inverse or U-shaped relationship between the HbA1c and death among patients with diabetes and heart failure especially among patients who were on insulin, sulphonylureas and thiazolidinediones [5]. Hypoglycemia among patients with diabetes is associated with an increasing health-related expenditure and healthcare resources utilization [6]. In the United state a single episode of hypoglycemia requiring a healthcare provider assistance is estimated at $1161 and 160 for direct and indirect costs respectively, a study conducted in Korea found that, the cost of a single hypoglycemic attack ranged from $17.28 to $1857.09 for secondary and tertiary hospitals. Thus hypoglycemia is a serious costly complication of diabetes and has a substantial economic burden. Furthermore, hypoglycemia is a principal obstacle to adherence to medications, poor glycemic control, and diabetes complications including microvascular complications [7–9], even a symptomatic Hypoglycemia is a cause of serious cardiac arrhythmias including long Q-T interval.

Lifestyle management including physical activity and adherence to a friendly healthier diet is an integral part of diabetes care. Nutrition therapy plays an integral role for every patient with diabetes mellitus [3].

Food insufficiency and unstable housing are associated with both diabetes risk, poor glycemic control, and hypoglycemia [10, 11].

Sudan is a vast country, the healthcare system is not well-established and centered mainly in large cities, diabetes care is lacking in rural areas if at all present with great difficulty in transportation. The total expenditure on health per capita is 282 US Dollars and may constitute 8.4% of gross domestic product [12]. Assessment of hypoglycemia risk and Hospital use in a country with limited resources and relating it to food insufficiency and poor housing condition is relevant and help the policy makers to directing the limited resources in the term of management and preventive strategies.

To our best of knowledge, this is the first study to assess the hypoglycemia risk, Hospital use and its related factors among patient with diabetes in Sudan. Thus we conducted this research to assess hypoglycemia risk, Hospital use, food insufficiency, and unstable housing condition among patients with type 2 diabetes in Sudan.

## Main text

### Materials and methods

#### Participants

This cross-sectional study was conducted at a diabetes center in Omdurman, Sudan during the period from June 2018 to September 2018. Alnour center is a public Clinic in Omdurman, Sudan accepting a wide range of patients under insurance cover and others who are not medically insured with minimal charges. The clinic was randomly chosen from twelve similar clinics. One hundred and fifty-nine consecutive patients with type 2 diabetes were approached. Those who were newly diagnosed (less than 1 year) were not included because they are expected to be younger [13]. Furthermore they may be taking only metformin, unlikely to be on insulin or sulphonylyureas [3], and may have lesser microvascular complications, so they are less prone to hypoglycemia.

#### Sample size calculation

The sample size was calculated using the following formula: n = Z^2^ P-Q/d^2^ where Z = 95% confidence (1.96), P = rate of diabetes mellitus Hospital admission in Sudan [14].

#### Questionnaire

All the participants were invited to sign a written informed consent, then interviewed using a structured questionnaire. The English version questionnaire has been previously validated to assess the risk of hypoglycemia Hospital use with good discrimination in both the internal and external validation [15]. The questionnaire was translated to Arabic by the researcher and an expert translator because it is newly developed [15] and not validated in Arabic. A facilitator (a co-patient) was present during the interview to assist if any difficulties raised during the interview to be sure that the patients understand the different components of the questionnaire. The questionnaire had two parts, the first part is the hypoglycemia hospital use score (six components) which asks about total number of prior hypoglycemia-related healthcare use episodes (three choices, zero, 1–2, and > 2), number of emergency-department encounters for any reason in the prior 12 months (two choices, < 2, ≥ 2 times), insulin use (yes/no),sulfonylurea use (yes/no), presence of severe or end-stage kidney disease (yes/no), age younger than 77 years (yes/no). Hypoglycemia risk calculator [16] was used to assess the risk of hypoglycemia.

The predicted 12-month risk of any hypoglycemia-related utilization was categorized as:

- High (> 5%).
- Intermediate (1%–5%).
- Low (< 1), with observed 12-month hypoglycemia-related utilization rates of 6.7%, 1.4%, and 0.2%, respectively.

Taking the wrong insulin or confusing the dose or units to be used and cigarettes smoking were also reported, the food insufficiency was tested using the following question: “are there times in the past 3 months when the food for you just did not last and there was no money to buy more?”, the second part constitutes the demographic data, access to exercise and a healthy diet. Unstable housing conditions as measured by having no money to buy (yes/no), or renting a house (yes/no) or moving two times/year (yes/no). Fasting plasma glucose and the glycated hemoglobin were taken from the patient’s records to assess the degree of glycemic control. The ethical committee of the Medical College, University of Tabuk, Saudi Arabia and Elnoor Polyclinic, Omdurman, Sudan approved the research.

#### Statistical analysis

The Statistical Package for Social Sciences (SPSS, version 20, New York) was used for data analysis. Binary logistic regression analysis was used to assess the relationship between hypoglycemia risk and different patient’s characteristics. A P-value of < 0.05 was considered significant.

### Results

#### Basic characteristics of the study group

There were 159 patients with diabetes (65.4% females), their ages ranged from 30 to 81 with a mean of 58.13 ± 9.96 years, the duration since the diagnosis of diabetes was 10.84 ± 8.41 years, the glycated hemoglobin was 9.98 ± 2.58, the mean fasting blood glucose was 178.05 ± 65.61 mg/dl, and 3.8% were cigarettes smokers. Table 1.

**Table 1:** Basic characteristics of the study group

Character	Mean ± SD
Age (years)	58.13 ± 9.96
Duration of diabetes (years)	10.84 ± 8.41
HbA1c	9.98 ± 2.58
Fasting plasma glucose (mg/dl)	178.05 ± 65.61
*Sex (no %)*	
Males	55 (34.6%)
Females	104 (65.4%)
Smoking (No %)	6 (3.8%)

#### Hypoglycemia risk score and its related factors

In the present study, access to a healthy diet and exercise were observed in 39% and 40.3% respectively, hypoglycemia necessitating hospital admission in the last 12 months was reported by 9.4%, emergency hospital visits due to high plasma glucose in the previous year was found in 27.3%, 27.3% were on insulin and nearly two-thirds were on sulphonylureas, 81.1% of the study sample were at low risk of hypoglycemia, 14.5% had intermediate risk, while 4.4% were at a high risk of hypoglycemia. Food insufficiency was reported in 66% of patients, and unstable housing was found in 15.1%. Table 2.

**Table 2:** Hypoglycemia risk score and its related factors

Character	No%
Adherence to a healthy diet	62 (39.0%)
Adherence to exercise	64 (40.3%)
Hypoglycemia	15 (9.4%)
Emergency Hospital visits	44 (27.3%)
Insulin use	44 (27.3%)
Sulphonylureas use	98 (61.6%)
*Hypoglycemia risk*	
Low	129 (81.1%)
Intermediate	23 (14.5%)
High	7 (4.4%)
Food insufficiency	105 (66.0%)
Unstable housing	24 (15.1%)

#### The relationship of hypoglycemia risk to, gender, food insufficiency, unstable housing conditions, HbA_1c_, fasting plasma glucose, and the duration since diabetes diagnosis

In the current study, no relationship was evident between hypoglycemia risk and gender, P-value = 0.287, 95% CI (0.003–5.849), food insufficiency, P-value = 0.122, 95% CI (0.392–2811.343), and unstable housing conditions, P-value = 0.999, 95% CI (0.000). The relationship of hypoglycemia risk to glycemic parameters and duration of diabetes are shown in Table 3.

**Table 3:** The relationship of hypoglycemia risk to, gender, food insufficiency, unstable housing conditions, HbA_1c_, fasting plasma glucose, and the duration since diabetes diagnosis

Variable	B	S.E.	Wald	df	Sig.	Exp (B)	95% CI for EXP (B)	
							Lower	Upper
Gender	− 2.098	1.972	1.132	1	.287	.123	.003	5.849
Food insufficiency	3.502	2.265	2.391	1	.122	33.195	.392	2811.343
Unstable housing	18.521	13,823.783	.000	1	.999	110,548,416.717	.000	–
HbA1c	.981	.820	1.430	1	.232	2.666	.534	13.306
FPG	− .051	.037	1.896	1	.168	.951	.884	1.022
DM duration	− .544	.316	2.967	1	.085	.581	.313	1.078

Logistic regression analysis

### Discussion

In the present study, the risk of hypoglycemia was high in 4.4%, intermediate in 14.5%, and low in 81.1% which is higher than a recent study conducted in the United States of America [17] and showed that 2.0%, 10.7%, and 87.3% were categorized as high, intermediate, and low risk, respectively, a study published in Korea [18] showed a prevalence of 0.96% for severe hypoglycemia which was lower than the current findings, the explanations could be the heterogeneity in hypoglycemia definition [19] and the high use of insulin in the present sample. The present findings necessitate the adoption of an interventional program to reduce the risk of hypoglycemia in our country by adherence to the guidelines and reduction of sulphonylureas and insulin prescription when not necessary to prevent its deleterious consequences in term of hospital use, cost, and cardiovascular mortality. The current data showed that 27.3% and 61.6% of patients were using insulin and sulphonylureas and were higher than a study conducted in Colombia [20] in which sulphonylureas use was found in 23.4%, and insulin in 20.7% this could in part explain the high risk of hypoglycemia in the current sample. Food insecurity has been shown to be associated with hypoglycemia and diabetes complications [21], in the present study, 66% of patients reported food insufficiency and was higher than rates reported in Kenya [22] (32%), and lower than a study conducted in three states in the United States [23] (84%), it is important to note that the US study was conducted among food pantry client, furthermore 50% of patients had mild food insecurity. The American Diabetes Association recommended physical activity and adherence to a healthy diet. In the present study, 59.7%, and 61% were not adherent to exercise and a diet rich in fruits and vegetables, the present findings were higher than Ghimire et al. [24] who reported that 41% and 46% of the participants were noncompliant to exercise advice and diet, another study [25] found that only 26% of individuals ate five or more servings of fruits and vegetables, and 33% met exercise recommendations and is in similarity to the present findings. It is prudent to raise awareness about the importance of physical activity and adherence to exercise in the management of diabetes mellitus. In the present study, no association was found between hypoglycemia risk, HbA1c, and fasting plasma glucose, the present findings were inconsistency with a previous study [26] who found that severe hypoglycemia in the previous year was common across all levels of glycemic control with the highest level observed among patients with near-normal or very poor glycemic control. Another study [17] concluded the association of poor glycemic control and severe hypoglycemia in contradiction to the present findings. Previous researches [22, 27] observed higher severe hypoglycemia among patients with foods insecurity especially among patients with household income below the national income, furthermore, the highest rate of hospital admission with hypoglycemia was commoner during the last week of the month due to exhaustion of food budget. In the current study, no relation between food insufficiency and hypoglycemia risk, a plausible explanation may be the difference in income and other socio-demographic characteristics or the small size of the present sample. In the current study, hypoglycemia risk and hospital use was not different among patients with unstable housing compared to those without in contradiction to a recent study [28] published in the US and found that unstable housing condition is common and is associated with increased risk of diabetes-related emergency department and inpatient use, a plausible explanation may be the small sample size in the current study. Malkani and Katwal [29] found that the duration of diabetes predicts hypoglycemia in type 1 diabetes, while young age predicted the same in type 2 diabetes. In the present study, no association was found between hypoglycemia risk and the duration of diabetes, the current finding of no differences between hypoglycemia risk and hospital use across gender is in line with Lipska et al. [30]. who concluded similar observation.

### Conclusion

Nearly one in five of patients with type 2 diabetes in the current sample were at risk of moderate/severe hypoglycemia (and thus healthcare utilization), and more than two-thirds had food insufficiency, furthermore, the majority of patients were not practicing exercise, and they were not adherent to a healthy diet. No relationship was found between hypoglycemia risk, HbA1c, fasting plasma glucose, food insufficiency, sex, unstable housing conditions, and diabetes duration. Incorporating hypoglycemia risk estimation in the holistic care of patients with diabetes is highly needed to reduce its fatal complications and emergency department and hospital admission and hence the cost of diabetes management. A comprehensive approach by the health care providers and adherence to the standard guidelines regarding lifestyle modifications and avoiding medications with high risk of hypoglycemia is highly recommended.

## Study limitations

The limitations of the study were the reliance of a self-reported interview which is prone to subjectivity, and the fact that the study was conducted at a single diabetes center, so generalization cannot be insured. The small size of the study sample is another limitation of the present study.

## Abbreviations

FPG: fasting plasma glucose
HbA_1c_: the glycated hemoglobin
SPSS: the statistical package for social sciences
